# Redox Status, Biochemical Parameters and Mineral Elements Content in Blood of Turkey Hens Fed a Diet Supplemented with *Yarrowia lipolytica* Yeast and Two *Bacillus Species*

**DOI:** 10.3390/ani10030459

**Published:** 2020-03-10

**Authors:** Anna Czech, Malwina Merska-Kazanowska, Zuzanna Całyniuk

**Affiliations:** Department of Biochemistry and Toxicology, Faculty of Animal Sciences and Bioeconomy, University of Life Sciences in Lublin, Akademicka 13, 20-950 Lublin, Poland; anna.czech@up.lublin.pl (A.C.); malwina.merska@interia.pl (M.M.-K.)

**Keywords:** turkey hens, *Yarrowia lipolytica* yeast, probiotic, blood, redox, biochemistry

## Abstract

**Simple Summary:**

Interest in *Yarrowia lipolytica* (YL) arises from the fact that it grows on the crude glycerol fraction derived from the production of diesel oil, so that it constitutes a link between biofuel by-products and animal feed. Studies have already been conducted on the use of *Yarrowia lipolytica* as a factor affecting the gut microbiota and fattening efficiency of turkeys, and the results have been promising. However, it has not been clarified whether *Yarrowia lipolytica* yeast can improve the availability of minerals as well as metabolic processes associated with redox reactions like *Saccharomyces cerevisiae* (SC). The research shows that, *Yarrowia lipolytica* yeast stimulates more effectively antioxidant processes than *Saccharomyces cerevisiae*, and also improves the absorption of minerals. Therefore, it may be a more effective additive than the widely used *Saccharomyces cerevisiae* yeast in turkey feed. In addition, the experiment confirmed that the antioxidant effect of fodder yeast (especially *Yarrowia lipolytica*) can be enhanced by combining it with a probiotic bacteria. Hence, the combined use of yeast with a *Bacillus* species can be recommended in cases of the stimulation of oxidative reactions (e.g., stress or infection).

**Abstract:**

The probiotic-prebiotic properties and chemical composition of *Yarrowia lipolytica* yeast (YL), predisposes it for use as a turkey feed additive. The aim of the study was to determine whether YL in the diet of turkeys would exert more beneficial effect by stimulating antioxidant reactions and increasing mineral availability than *Saccharomyces cerevisiae* (SC). An additional aim of the study was to test whether the addition of a probiotic bacteria to feed containing *Yarrowia lipolytica* or *Saccharomyces cerevisiae* yeast would enhance their effect. The study was conducted on turkeys from seven to 112 days of age to determine the effects of a 3% addition of YL to the diet, as an alternative to the standard SC. It was analysed whether the use of a probiotic (*Bacillus licheniformis*, *Bacillus subtilis*) together with yeast would be more effective. Both the yeast (YL or SC) and the probiotic stimulated antioxidant mechanisms (increased CAT; reduced MDA), but the addition of SC was less effective. The inclusion of YL in the feed increased the plasma content of Ca, Mg, Cu, Zn and Fe in the blood turkeys and lowered the content of cholesterol and triacylglycerols. The combined use of yeast (YL or SC) with a probiotic enhances the antioxidant effect while inhibiting of lipid peroxidation. The combined use of yeast with a probiotic can be recommended in cases of stimulation of oxidative reactions (e.g., stress or infection)

## 1. Introduction

*Saccharomyces cerevisiae* (SC) is a yeast species widely used in poultry production as a natural growth stimulant and a source of complete protein [1]. In addition, the β-glucans and antioxidants contained in the cell walls of *Saccharomyces cerevisiae* can modulate gastrointestinal processes, thus exerting a positive effect on gut microbiota [2], stimulating nutrient absorption, minimizing the production of oxygen free radicals, and neutralizing their effects [3]. In recent years, research has been conducted on the use of *Yarrowia lipolytica* yeast (YL) in feed for poultry as an alternative to the commonly used *Saccharomyces cerevisiae*. Interest in *Yarrowia lipolytica* arises from the fact that it grows on the crude glycerol fraction derived from the production of diesel oil [4], so that it constitutes a link between biofuel by-products and animal feed [5]. Studies conducted thus far indicate that the nutritional value of *Yarrowia lipolytica* makes it suitable for use in animal diets [6,7]. Compared to *Saccharomyces cerevisiae*, it has a higher content of protein (approx. 45%), with high biological value due to its well-balanced amino acid composition. *Yarrowia lipolytica* also has higher content of macro- and microelements than *Saccharomyces cerevisiae* (10% more Na^+^ and about 30% more Fe^2+^) and of amino acids, on average by 18% in the case of tyrosine, lysine, threonine or leucine. In addition, *Yarrowia lipolytica* can more effectively accumulate ions of divalent metals such as iron, copper and zinc, thus improving their bioavailability. Unlike *Saccharomyces cerevisiae*, they also have unique physical properties (aroma and sweet taste) and contain many valuable enzymes and large amounts of B vitamins, choline, biotin and niacin. The chemical composition and physical properties of *Yarrowia lipolytica* make it an interesting feed additive. Studies have already been conducted on the use of *Yarrowia lipolytica* as a factor affecting the gut microbiota and fattening efficiency [8,9,10], and the results have been promising. However, it has not been clarified whether *Yarrowia lipolytica* yeast, like *Saccharomyces cerevisiae*, can improve the availability of minerals as well as metabolic processes associated with redox reactions.

Many studies indicate that the effect of fodder yeast can be enhanced by using it in combination with probiotics [9,11]. As the addition of a probiotic bacteria to feed containing *Saccharomyces cerevisiae* cell wall has been shown to stimulate body weight and mineral availability in broilers [12] we decided to test it together with the addition with YL. We hypothesized that the addition of YL yeast to turkey feed together with a probiotic can inhibit oxidative processes and thus improve utilization of nutrients and minerals. This can be of great importance for the development of new principles of disease prevention in turkey farming.

Therefore, the aim of the study was to determine whether *Yarrowia lipolytica* yeast in the diet of turkeys would exert a beneficial effect by stimulating antioxidant reactions and increasing mineral availability similarly to the commonly used species *Saccharomyces cerevisiae*. An additional aim of the study was to test whether the addition of a probiotic to feed containing *Yarrowia lipolytica* or *Saccharomyces cerevisiae* yeast would enhance their effect.

## 2. Material and Methods

### 2.1. Ethics Statement

The experimental procedure was approved by the Local Ethics Commission for Experiments with Animals in Lublin (approval no. 19/12).

### 2.2. Animals and Experimental Design

The experiment was carried out on 480 BIG 6 turkey hens randomly divided into 6 groups of 80 (5 repetitions of 16 turkeys reared to the age of 112 days). During the experiment, birds from all groups received ad libitum complete balanced feeds according to the recommendations of NRC [13] for each rearing period (Starter: 1–4 weeks; Grower 1: 5–8 weeks; Grower 2: 9–12 weeks; Finisher: 13–16 weeks). All feeds were balanced in terms of nitrogen and metabolic energy.

Birds from all experimental groups received feed based on wheat (45.3%–47.5% for all rearing periods) and soybean meal (19.5%–40.5% for all rearing periods), and additionally triticale (10%—Grower 2 and Finisher), rapeseed cake (4%—Grower 1; 6%—Grower 2; 9%—Finisher) and potato protein (5%—Starter). Soybean oil was added to all feeds. The feed mixtures composition and chemical composition of feed mixtures are presented in Czech et al. [8].

Birds from the control group (C) and group CP were fed standard feeds without the addition of yeast, but group CP additionally received a probiotic, which was a mixture of *Bacillus licheniformis*—1.6 × 10^9^ CFU/g and *Bacillus subtilis*—1.6 × 10^9^ CFU/g, in the amount of 0.05%. Groups Y and YP received feed containing 3% *Yarrowia lipolytica* fodder yeast (2 × 10^10^ CFU/g—Scotan S.A, Poland), and for the YP group the probiotic was additionally added to the feed in the amount of 0.05%. Similarly, in groups S and SP, the turkeys received feed with 3% *Saccharomyces cerevisiae* (3 × 10^10^ CFU/g—Inter S^®^) fodder yeast, and for the SP group the probiotic was additionally added to the feed in the amount of 0.05 kg per tonne of feed (Table 1). The optimal dosage of the yeasts (3%) was based on results obtained in a previous study [14].

### 2.3. Experimental and Laboratory Procedures

At the end of the experiment, at 112 d of age, blood was drawn from the wing vein of 10 turkeys from each group. The blood was collected after 8 h without feed.

The biochemical parameters and mineral content were determined in blood plasma, which was obtained by centrifuging whole blood at 3000× *g* for 10 min. Analyses were performed 3–4 h after the blood was collected. The blood was stored at 4 °C.

Test kits developed by Cormay (Lublin, Poland) were used to determine the content of total protein (TP, Liquick Cor-TOTAL PROTEIN 60, Lublin, Poland), glucose (GLU, Liquick Cor-GLUCOSE 60, Lublin, Poland), uric acid (UA, Liquick Cor-UA 60, Lublin, Poland), urea (UREA, Liquick Cor-UREA 60, P Lublin, Poland), creatinine (CREAT, Liquick Cor-CREA ENZYMATIC 60, Lublin, Poland), and bilirubin (BIL, Liquick Cor-BIL Total, Lublin, Poland), triglycerides (TG, Liquick Cor-TG 30, Lublin, Poland), total cholesterol (TCH, Liquick Cor-CHOL 60, Lublin, Poland), and high-density lipoprotein cholesterol (HDL, Liquick Cor-HDL, Lublin, Poland). The percentage of HDL in relation to total cholesterol (% HDL) and low-density lipoprotein cholesterol (LDL) were calculated as well. Ready-to-use test kits were used to determine the activity of selected enzymes: alanine aminotransferase (ALT, Liquick Cor-ALAT 60, Lublin, Poland), aspartate aminotransferase (AST, Liquick Cor-ASAT 60, Lublin, Poland), alkaline phosphatase (ALPv, Liquick Cor-ALP 60, Lublin, Poland), and lactate dehydrogenase (LDH, Liquick Cor-LDH 30, Lublin, Poland). The plasma content of phosphorus (P, Liquick Cor-PHOSPHORUS 30, Lublin, Poland), calcium (Ca, Liquick Cor-CALCIUM ARSENOZO 60, Lublin, Poland), magnesium (Mg, Liquick Cor-MG, Lublin, Poland), iron (Fe, Liquick Cor-FERRUM, Lublin, Poland), zinc (Zn, BioMaxima, Lublin, Poland) and copper (Cu, BioMaxima, Lublin, Poland) was determined by the colorimetric method.

The turkey blood plasma was also used for spectrophotometric measurement of superoxide dismutase (SOD) activity was determined, with an adrenaline assay according to Heikkila [15]. SOD activity is determined by measuring the rate of auto-oxidation of adrenaline at 30 °C based on the increase of absorbance at 320 nm (which corresponds the increase in concentration of various products of adrenaline oxidation). Catalase (CAT) activity was determined according to Clairborne [16]. The analysis was based on measurement of the rate of substrate (hydrogen peroxide) decomposition catalysed by this enzyme.

The total antioxidant potential of the plasma (FRAP) was recorded following Benzie and Strain [17]. The method utilized the antioxidant capacity to reduce Fe^3+^ to Fe^2+^, which forms a coloured complex with 2,4,6 tripyridyltriazine (TPTZ) present in the solution. The increase in absorbance of the TPTZ-Fe^2+^ complex is proportional to amount of antioxidant in the test tube. Vitamin C content in the blood plasma was estimated according to Omaye et al. [18].

The plasma level of malondialdehyde (MDA) was determined according to Salih et al. [19], and lipid hydroperoxide concentration (LOOH) as described by Gay and Gebicki [20].

### 2.4. Statistical Analysis

Statistical calculations of the studied traits were performed by two-factorial model with interaction, taking into account the influence of the feeding group described in Table 2. The calculations were made using general linear models (procedure GLM) in SAS 9.4 software (SAS Institute, Cary, NC, USA). The significance of differences between means was determined using Tukey’s test.

## 3. Results

A more complete evaluation of YL *vs* SC efficacy on the results of production and histopathological evaluation of the gastrointestinal tract was presented in the study by Czech et al. [8].

Table 2 presents the values of biochemical and antioxidant parameters and the content of minerals in the plasma of turkeys receiving yeast (*Yarrowia lipolytica* or *Saccharomyces cerevisiae*) and/or a probiotic (*Bacillus licheniformis* and *Bacillus subtilis*) in their feed.

A significant increase in TP, UA, UREA, and CRET content, as well as SOD and CAT activity, FRAP value, and vitamin C content was observed in the plasma of turkeys receiving feed with *Yarrowia lipolytica* yeast relative to the groups without yeast. The addition of *Yarrowia lipolytica* yeast increased the concentrations of magnesium, copper, zinc and iron compared to the groups not receiving *Yarrowia lipolytica*, while the addition of *Saccharomyces cerevisiae* significantly increased the plasma concentration of phosphorus and iron (Table 2 and Table 3).

The addition of *Saccharomyces cerevisiae* increased the content of UREA, CREAT and BIL, as well as SOD, CAT and ALP activity, relative to the groups without yeast.

A significant decrease in the plasma content of malondialdehyde (MDA) was recorded in the groups of turkeys receiving *Yarrowia lipolytica* or *Saccharomyces cerevisiae* (Table 2 and Table 3).

The addition of *Yarrowia lipolytica* or *Saccharomyces cerevisiae* caused a significant reduction in the plasma content of total cholesterol and its HDL and LDL fractions compared to birds not receiving a yeast supplement. It should be noted, however, that the addition of *Yarrowia lipolytica* or *Saccharomyces cerevisiae* yeast significantly increased % HDL relative to the group not receiving a yeast additive. Plasma content of TG was significantly lower in turkeys receiving the *Saccharomyces cerevisiae* supplement (Table 3).

The addition of a probiotic (*Bacillus licheniformis* and *Bacillus subtilis*) to turkey feed significantly increased the content of UA, CHOL, HDL and LDL in the blood plasma, activity of CAT and ALT, and total antioxidant potential (FRAP) relative to the group that did not receive this additive. The content of UREA, Mg, Cu and TG as well as AST activity in the plasma of turkeys whose feed was supplemented with the probiotic decreased significantly relative to groups not receiving the probiotic (Table 2, Table 3 and Table 4).

The plasma of turkeys receiving the *Saccharomyces cerevisiae* supplement had a significantly lower FRAP value, UA, Mg, Cu, Zn, and TP content and LDH activity than that of turkeys receiving *Yarrowia lipolytica*. Significantly lower ALP and ALT activity were noted in the plasma of turkeys whose feed contained *Yarrowia lipolytica* compared to birds receiving *Saccharomyces cerevisiae*.

The addition of the probiotic to *Yarrowia lipolytica* yeast (group YP) caused an increase in the content of TP, GLU, UA and Ca and in LDH activity compared to other groups (Table 3 and Table 4).

Comparison of the combined used of the probiotic and *Yarrowia lipolytica* yeast with the combined use of the probiotic and *Saccharomyces cerevisiae* (YP *vs* SP) shows significantly higher content of TP, UA, GLU, CREAT, CHOL, HDL, LDL and MDA as well as higher AST and LDH activity in the blood plasma of birds from the YP group, while ALP and ALT activity and content of P and LOOH were lower (*p ≤* 0.05) (Table 2, Table 3 and Table 4).

## 4. Discussion

According to Aluwong et al. [3], owing to the ability to maintain a reducing environment in the body, yeast can indirectly contribute to the neutralization of free radicals, transforming them into less active derivatives. This delays or even inhibits oxidation processes. Yeasts are also recognized as inhibitors of oxidation, hence their significant role in the fight to restore and maintain homeostasis in the body [21]. A measure of redox status is the balance between oxidative factors such as lipid peroxidation products (malondialdehyde or peroxide radicals) and endogenous and exogenous antioxidant substances [3]. In the present study, the use of *Yarrowia lipolytica* or *Saccharomyces cerevisiae* yeast resulted in a significant increase in catalase (CAT) and superoxide dismutase (SOD) activity, which may indicate their role in the activation of antioxidant enzymes and reduction of peroxidation products [9]. According to Aluwong et al. [3], an increase in antioxidant enzyme activity may be associated with increased resistance to colonization and susceptibility to environmental pathogens, which can be achieved by including fodder yeast in poultry diets.

It was confirmed in the experiment conducted by Czech et al. [8], as the presence of *Yarrowia lipolytica* yeast minimized the amount of pathogens in the gastrointestinal tract, which resulted in the improvement of intestinal integrity (prolongation of intestinal villi length by 50% and improvement of V/IC ratio by 50%, on average). It also had a direct impact on the improvement of production effects (improvement of FI and reduction of FCR by about 4%) [8].

The relationships observed in the present study are consistent with reports by Milinkovic-Tur et al. [22], who noted increased SOD and CAT activity in the myocardium of broiler chickens receiving feed containing yeast. Moreover, Ognik and Krauze [23] have shown that mannan oligosaccharides found in the yeast cell wall stimulate antioxidant protection mechanisms and improve the growth rate of birds. This indicates that yeast can protect the gastrointestinal tract by other means than removing unwanted bacteria. Kogan et al. [24] also suggest that β-glucans in yeast cell walls may exhibit antioxidant activity, and Krizkova et al. [25] report that these properties are already present in the feed. This is confirmed by the work of Petrova et al. [26], which has shown that there are two types of catalase in the yeast cell wall: type A (atypical, peroxisomal catalase) and type T (typical, cytoplasmic catalase).

The activity of catalase and other antioxidant enzymes is supported by other components of antioxidant defence, including low-molecular-weight antioxidants such as bilirubin, uric acid, urea, glutathione, and active forms of vitamins E and C, and depends on the presence of dissociated cations of divalent elements, such as i.e. Cu^2+^, Zn^2+^, Mn^2+^, Fe^2+^, and Se^2+^ [3]. In our experiment, the addition of *Yarrowia lipolytica* yeast increased the plasma concentration not only of low-molecular-weight antioxidants (such as UA, UREA, and CREAT) but also micronutrients (such as Mg, Cu, Zn, and Fe), which translated into maintenance of antioxidant enzyme activity. Catalase has a haem group with a centrally located iron atom (Fe^3+^), so the increase in iron content in the plasma of turkeys receiving feed supplemented with *Yarrowia lipolytica* or *Saccharomyces cerevisiae* may have contributed to the increased activity of this enzyme. Higher levels of SOD activity, on the other hand (observed mainly in the groups receiving the *Yarrowia lipolytica*), were correlated with an increase in the concentration of copper ions, which are present in its catalytic centre and react with superoxide anion radical [27], and also with an increased amount of zinc ions, which stabilize the tertiary structure of the enzyme [21]. This may be confirmed by the research of Ognik and Krauze [23], in which an increase in the content of divalent micronutrients in turkey blood plasma was accompanied by intensification of pro-oxidative processes. The combined effect of SOD and CAT leads to the safe removal of lipid peroxides, which was reflected in reduced concentration of lipid peroxidation products such as malondialdehyde. This was particularly evident in turkeys receiving the probiotic (*Bacillus licheniformis* and *Bacillus subtilis*) together with *Yarrowia lipolytica* or *Saccharomyces cerevisiae* yeast. These results correspond to findings by Popović et al. [28], who observed a significant reduction in MDA in the blood of chickens receiving feed supplemented with a probiotic in combination with a prebiotic (synbiotic). According to Li et al. [29], supplementing poultry feed with mannan oligosaccharides and β-glucans reduces accumulation of the lipid oxidation end product (MDA) in the tissues of broiler chickens and improves the oxidative status of weaned piglets [30].

Uric acid present in the blood plasma is another factor influencing SOD activity, by preventing the inactivation of SOD by hydrogen peroxide. The urate radical formed in the reaction is rapidly regenerated to uric acid in a reaction with L-ascorbic acid [27]. This was observed in our experiment, as the plasma of turkeys receiving feed supplemented with *Yarrowia lipolytica* or *Saccharomyces cerevisiae*, in addition to an increase in SOD activity, also had a higher concentration of uric acid. The increase in uric acid content was correlated with an increase in total antioxidant capacity (FRAP), which is indicative of better protection of cells and tissues against the toxic effects of reactive oxygen species (ROS) [31]. The increased FRAP in the turkey plasma was also accompanied by an increase in the content of creatinine and other FRAP components, such as vitamin C, but mainly micronutrients such as iron, copper and zinc.

The FRAP value is also affected by the presence in proteins of sulfhydryl groups (SH), which constitute about 10% of FRAP. An increased FRAP value could suggest better absorption of sulphur-containing amino acids from feed containing *Yarrowia lipolytica*. Studies on chickens [32] and turkeys [33] indicate that higher methionine levels in poultry feed have an antioxidant effect. In addition, it should be noted that the turkeys receiving these yeasts had a significantly higher plasma content of total protein. The increase in blood protein levels could also be due to the presence of chitin, mannan or glucan, derivatives of the cell wall of yeast, which not only stimulate the immune system, but also increase globulin levels [34].

Inhibition of oxidative processes through the addition of fodder yeast or a probiotic may be reflected in lipid metabolism processes. The addition of fodder yeast significantly changed the value of blood lipid components. In turkeys receiving feed with *Yarrowia lipolytica* or *Saccharomyces cerevisiae* yeast, there was a significant reduction in total and LDL cholesterol relative to the control group. The results correspond to the findings of numerous studies [3]. The effect was likely linked to the beneficial effects of yeast on the gut microbiota, which may have resulted in better utilization of lipid components (synthesis of bile acids and steroid hormones) [2]. Furthermore, lipids, mainly LDL, are protected by antioxidants, and when low-molecular-weight antioxidants are lacking in the body under stress conditions, they can be oxidized, or different classes of lipoproteins can exchange lipids, because HDL contains more lipid pro-oxidants than LDL. Therefore, HDL lipids are oxidized more easily. This may suggest that the administration of yeast cell walls could stimulate a high level of low-molecular-weight antioxidants [35]. This may be demonstrated by the higher urea and FRAP values in the plasma of the experimental turkeys receiving feed with *Yarrowia lipolytica* or *Saccharomyces cerevisiae* yeast, which protects the organism from oxygen free radicals during lipid peroxidation.

## 5. Conclusions

*Yarrowia lipolytica* yeast more effectively stimulates antioxidant processes than *Saccharomyces cerevisiae*, and also improves the absorption of minerals. Therefore, it may be a more effective additive than the widely used *Saccharomyces cerevisiae* yeast in turkey feed. In addition, the experiment confirmed that the antioxidant effect of fodder yeast (especially *Yarrowia lipolytica*) can be enhanced by combining it with a probiotic. Hence, the combined use of yeast with a probiotic can be recommended in cases of the stimulation of oxidative reactions (e.g., stress or infection).

## Figures and Tables

**Table 1:** Experimental design.

Feeding Group		Yeast		
		None (Control)	3% *Yarrowia lipolytica*	3% *Saccharomyces crevisiae*
Probiotic	none (control)	C	Y	S
	*Bacillus licheniformis* (1.6 × 10^9^ CFU/g) and *Bacillus subtilis* (1.6 × 10^9^ CFU/g) —0.05%	CP	YP	SP

**Table 2:** Results of statistical analysis of pro- and antioxidant blood plasma parameters in turkey hens.

Feeding Groups	Probiotic		Yeast			Probiotic × Yeast ^1^						
Trait	–	+	none	Y	S	C (n = 10)	CP (n = 10)	Y (n = 10)	YP (n = 10)	S (n = 10)	SP (n = 10)	SE
**Antioxidants ^2^**												
SOD; U L^−1^	37.66	39.61	37.37 ^b^	39.53 ^a^	39.01 ^a^	34.92 ^b^	39.82 ^ab^	38.76 ^ab^	40.35 ^a^	39.36 ^ab^	38.65 ^ab^	1.16
CAT; U L^−1^	4.15 ^b^	4.70 ^a^	4.14 ^b^	4.68 ^a^	4.47 ^a^	3.75 ^b^	4.52 ^ab^	4.18 ^ab^	5.17 ^a^	4.52 ^ab^	4.41 ^ab^	0.321
FRAP; µmol L^−1^	33.62 ^b^	38.29 ^a^	35.25 ^b^	38.28 ^a^	34.34 ^b^	33.26 ^bc^	37.24 ^ab^	36.15 ^ab^	40.41 ^a^	31.45 ^c^	37.23 ^ab^	1.71
Vitamin C; mg L^−1^	1.13	1.08	0.983 ^b^	1.23 ^a^	1.09 ^ab^	0.815 ^b^	1.15 ^ab^	1.32 ^a^	1.14 ^ab^	1.24 ^ab^	0.945 ^ab^	0.108
**Prooxidants ^2^**												
LOOH; µmol L^−1^	2.43	2.13	2.30	2.18	2.36	2.37	2.23	2.55	1.81	2.37	2.34	0.238
MDA; µmol L^−1^	1.09	0.973	1.19 ^a^	0.961 ^b^	0.945 ^b^	1.26 ^a^	1.14 ^ab^	0.941 ^bc^	0.984 ^b^	1.09 ^b^	0.802 ^c^	0.053

^a–^^c^: means in rows with different letters differ significantly at *p* ≤ 0.05; ^1^ C: control; CP: addition of probiotic to feed; Y: 3% share of *Yarrowia lipolytica* in feed; YP: addition of probiotic and 3% share of *Yarrowia lipolytica* in feed; S: 3% share of *Saccharomyces cerevisiae* in feed; SP: addition of probiotic and 3% share of *Saccharomyces cerevisiae* in feed. ^2^ SOD: superoxide dismutase, CAT: catalase, FRAP: total antioxidant potential of the plasma, LOOH: lipid hydroperoxide, MDA: malondialdehyde.

**Table 3:** Results of statistical analysis of biochemical blood plasma parameters in turkey hens.

Feeding Groups	Probiotic		Yeast			Probiotic × Yeast ^1^						
Trait	–	+	none	Y	S	C (n = 10)	CP (n = 10)	Y (n = 10)	YP (n = 10)	S (n = 10)	SP (n = 10)	SE
**Biochemical parameters ^2^**												
TP; g L^−1^	37.82	40.86	37.63 ^b^	42.63 ^a^	37.77 ^b^	35.72 ^c^	39.56 ^b^	38.51 ^b^	46.74 ^a^	39.24 ^b^	36.29 ^c^	0.514
GLU; mmol L^−1^	15.28	15.68	15.39	15.88	15.18	15.01 ^cd^	15.77 ^b^	15.22 ^bc^	16.53 ^a^	15.62 ^bc^	14.74 ^d^	0.298
UA; µmol L^−1^	194.8 ^b^	250.9 ^a^	213.5 ^b^	261.5 ^a^	193.6 ^b^	156.9 ^d^	270 ^b^	218.3 ^c^	304.8 ^a^	209.2 ^c^	177.9 ^d^	6.75
UREA; mmol L^−1^	0.579 ^a^	0.477 ^b^	0.410 ^b^	0.562 ^a^	0.619 ^a^	0.546 ^bc^	0.275 ^d^	0.505 ^c^	0.619 ^ab^	0.684 ^a^	0.554 ^bc^	0.035
CREAT; µmol L^−1^	28.87	25.45	22.17 ^b^	29.97 ^a^	29.35 ^a^	20.94 ^d^	23.4 ^c^	32.43 ^a^	27.51 ^b^	33.25 ^a^	25.45 ^c^	0.733
BIL; µmol L^−1^	10.11	12.09	9.24 ^b^	10.88 ^ab^	13.18 ^a^	7.59 ^c^	10.88 ^b^	8.38 ^c^	13.38 ^a^	14.36 ^a^	12.00 ^ab^	0.532
**Enzyme activity (U L^-1^) ^2^**												
ALP	943.9	963.3	971.4 ^a^	918.9 ^b^	970.6 ^a^	988 ^a^	954.7 ^b^	893.4 ^c^	944.4 ^b^	950.3 ^b^	990.9 ^a^	14.21
ALT	5.63 ^b^	6.65 ^a^	6.43	5.85	6.15	6.43 ^b^	6.42 ^b^	5.36 ^c^	6.39 ^b^	5.16 ^c^	7.13 ^a^	0.230
AST	161.2	147.3	164.4	155.1	143.2	184.7 ^a^	144.1 ^b^	152.5 ^b^	157.6 ^b^	146.3 ^b^	140.1 ^b^	7.22
LDH	483.8	487.3	503.6 ^a^	525.2 ^a^	427.9 ^b^	509.9 ^ab^	497.3 ^ab^	512.5 ^ab^	537.9 ^a^	429.0 ^b^	426.7 ^b^	6.69
**Lipid blood parameters ^2^**												
TCH; mmol L^−1^	2.90 ^b^	3.23 ^a^	3.37 ^a^	2.88 ^b^	2.94 ^b^	3.47 ^a^	3.27 ^a^	2.88 ^b^	2.87 ^b^	2.34 ^c^	3.54 ^a^	0.107
HDL; mmol L^−1^	1.74 ^b^	1.91 ^a^	1.95 ^a^	1.83 ^b^	1.71 ^b^	1.99 ^a^	1.90 ^ab^	1.83 ^b^	1.82 ^b^	1.41 ^c^	2.01 ^a^	0.072
LDL; mmol L^−1^	0.330 ^b^	0.433 ^a^	0.455 ^a^	0.199 ^b^	0.388 ^ab^	0.453 ^a^	0.459 ^a^	0.297 ^b^	0.303 ^b^	0.241 ^b^	0.536 ^a^	0.052
TG; mmol L^−1^	0.460 ^a^	0.379 ^b^	0.474 ^a^	0.397 ^ab^	0.375 ^b^	0.580 ^a^	0.36 ^b^	0.410 ^b^	0.39 ^b^	0.401 ^b^	0.350 ^b^	0.046
% HDL	60.94	59.68	58.02 ^b^	63.97 ^a^	58.95 ^ab^	57.87	58.16	64.21	63.73	60.74	57.15	3.42
TCH/HDL	1.67	1.71	1.76 ^a^	1.59 ^b^	1.72 ^ab^	1.76	1.75	1.59	1.60	1.67	1.77	0.100

^a–^^d^ means in rows with different letters differ significantly at *p* ≤ 0.05; ^1^ C: control; CP: addition of probiotic to feed; Y: 3% share of *Yarrowia lipolytica* in feed; YP: addition of probiotic and 3% share of *Yarrowia lipolytica* in feed; S: 3% share of *Saccharomyces cerevisiae* in feed; SP: addition of probiotic and 3% share of *Saccharomyces cerevisiae* in feed. ^2^ TP: total protein, GLU: glucose, UA: uric acid, UREA: urea, CREAT: creatinine, BIL: bilirubin, ALT: alanine aminotransferase, AST: aspartate aminotransferase ALP: alkaline phosphatase, LDH: lactate dehydrogenase, TG: triacylglycerols, TCH: total cholesterol, HDL: high-density lipoprotein cholesterol, LDL: low-density lipoprotein cholesterol.

**Table 4:** Results of statistical analysis of mineral elements blood plasma in turkey hens.

Feeding Groups	Probiotic		Yeast			Probiotic × Yeast ^1^						
Trait	–	+	none	Y	S	C (n = 10)	CP (n = 10)	Y (n = 10)	YP (n = 10)	S (n = 10)	SP (n = 10)	SE
Phosphorus; mmol L^−1^	1.89	1.87	1.81 ^b^	1.86 ^b^	1.97 ^a^	1.87 ^b^	1.74 ^c^	1.88 ^b^	1.84 ^b^	1.92 ^ab^	2.02 ^a^	0.056
Calcium; mmol L^−1^	2.40	2.65	2.44	2.58	2.56	2.37 ^b^	2.51 ^b^	2.38 ^b^	2.77 ^a^	2.44 ^b^	2.68 ^a^	0.062
Magnesium; mmol L^−1^	0.979 ^a^	0.862 ^b^	0.917 ^b^	0.984 ^a^	0.861 ^b^	0.975 ^b^	0.859 ^c^	1.11 ^a^	0.858 ^c^	0.851 ^c^	0.870 ^c^	0.029
Copper; µmol L^−1^	6.43 ^a^	5.86 ^b^	5.92 ^b^	6.58 ^a^	5.94 ^b^	6.51 ^ab^	5.32 ^d^	6.82 ^a^	6.34 ^bc^	5.95 ^c^	5.93 ^c^	0.192
Zinc; µmol L^−1^	19.53	18.74	18.02 ^b^	20.99 ^a^	18.41 ^b^	18.34 ^c^	17.69 ^d^	22.73 ^a^	19.24 ^b^	17.51 ^d^	19.33 ^b^	0.278
Iron; µmol L^−1^	31.31	31.43	28.74 ^b^	34.26 ^a^	31.11 ^ab^	29.22 ^b^	28.25 ^b^	33.84 ^a^	34.68 ^a^	30.87 ^ab^	31.35 ^ab^	1.58

^a–^^d^ means in rows with different letters differ significantly at *p* ≤ 0.05; ^1^ C: control; CP: addition of probiotic to feed; Y: 3% share of *Yarrowia lipolytica* in feed; YP: addition of probiotic and 3% share of *Yarrowia lipolytica* in feed; S: 3% share of *Saccharomyces cerevisiae* in feed; SP: addition of probiotic and 3% share of *Saccharomyces cerevisiae* in feed.
